# Evaluation of Macro- and Micro-Geometry of Models Made of Photopolymer Resins Using the PolyJet Method

**DOI:** 10.3390/ma17174315

**Published:** 2024-08-30

**Authors:** Paweł Turek, Anna Bazan, Grzegorz Budzik, Tomasz Dziubek, Łukasz Przeszłowski

**Affiliations:** Faculty of Mechanical Engineering and Aeronautics, Rzeszów University of Technology, 35-959 Rzeszów, Poland; abazan@prz.edu.pl (A.B.); gbudzik@prz.edu.pl (G.B.); tdziubek@prz.edu.pl (T.D.); lprzeszl@prz.edu.pl (Ł.P.)

**Keywords:** additive manufacturing, photopolymer resins, accuracy, macro-geometry, micro-geometry, optical measurement

## Abstract

Additive manufacturing (AM) techniques are among the fastest-growing technologies for producing even the most geometrically complex models. Unfortunately, the lack of development of metrology guidelines for these methods, related to dimensional and geometry accuracy and surface roughness, significantly limits the commercialization of finished products manufactured using these methods. This paper aims to evaluate the macro- and micro-geometry of models manufactured using the PolyJet method from three types of photopolymer resins: Digital ABS Plus, RGD 720, and Vero Clear. For this purpose, test parts were designed and then manufactured on an Object 350 Connex3 3D printer. The Atos II Triple Scan optical system and the InfiniteFocusG4 microscope were used to evaluate macro- and micro-geometry, respectively. For both systems, measurement procedures were developed to obtain statistical results for evaluating geometric accuracy and surface roughness parameters. In the case of macro-geometry, for Digital ABS Plus and Vero Clear materials, 50% of the central deviations (between first quartile Q1 and third quartile Q3) lie within the range (−0.06, 0.03 mm) and for RGD 720 material within the range (−0.08, 0.01 mm). For micro-geometry, the arithmetic mean height (Sa) values for the Digital ABS Plus and Vero Clear samples were approximately 1.6 and 2.0 µm, respectively, while for RGD 720, it was 15.9 µm. The total roughness height expressed by reduced peak height (Spk) + core height (Sk) + reduced dale depth (Svk) for the Digital ABS Plus and Vero Clear samples was approximately 9.1 and 10.5 µm, respectively, while for the RGD 720, it was 101.9 µm.

## 1. Introduction

The product of each component involves specific technological processes [1,2]. These processes define the geometry, dimensions, and stereometry of the final model, and they impact the microstructure and mechanical and physicochemical properties [3,4]. The result of this technological process is the outer layer of the component, known as the surface texture [5]. Additive methods have become increasingly popular for manufacturing physical models [6,7]. Using additive techniques involves applying the material in layers until the complete model is obtained [8]. Models produced using additive manufacturing (AM) methods are widely used in aerospace [9,10,11], automotive [12,13], medical [14,15], dental [16] industries, and others. As a result, the manufactured models have specific quality requirements, including dimensional and geometric accuracy [17], and surface roughness [18].

Due to several factors, none of the additive techniques are currently dominant in industrial applications. When assessing the accuracy of the manufactured product, errors in dimensions and geometry occur during manufacturing [19,20], and there is a noticeable change in surface roughness [21,22]. The resulting differences are influenced by various factors, mainly related to the manufacturing parameters and post-processing treatment. The layer thickness used has the most significant influence on reproduction accuracy [6,8,23], and it is influenced by the design parameters of the 3D printer, software parameters, and applied manufacturing modes. The layer thickness and the direction of the model build affect the accuracy of the geometry reproduction [24,25], especially for freeform surfaces or surfaces inclined at an angle other than 90° to the direction of subsequent layers [26]. The support structure in the model manufacturing process also affects the quality of the model surface representation, mainly influenced by the type of post-processing treatment adopted [27,28,29]. As a result, the model’s surface often differs from the designer’s assumptions after being cleaned of the supporting material. The accuracy of models is greatly influenced by the choice of model material [6,9]. According to Wohlers’ 2021 report, nearly half of AM service providers currently offer polymeric materials for 3D printing, and around 29% also provide polymers in addition to other materials like metals and ceramics [30]. As a result, over 80% of the AM market is focused on polymer materials. This is because polymeric materials are increasingly being used in place of traditional engineering materials and can be utilized to create machine and mechanism components and functional models [12,31,32].

Currently, there is an increasing trend in using the PolyJet method to produce models for the medical industry, especially the dental industry [33,34], as well as injection molds and model sets for silicone molds used in short production runs [35,36,37,38]. This is because parts manufactured using the PolyJet method from photopolymer materials are accurate and have a smooth surface finish comparable to parts made by injection molding [39]. One distinguishing feature of the PolyJet method is the ability to produce thin walls [40]. However, more work is needed to develop design assumptions for the dimensional and geometrical accuracy and surface roughness of models manufactured using the PolyJet technique. This complexity significantly affects the commercialization of products fabricated using this method. Considering the ISO/ASTM 52902 standard, it includes developed 3D CAD models for verifying the accuracy of 3D printers [41]. The standard also guides the selection of measuring tools to carry out metrological verification of manufactured models. However, ISO/ASTM 52902 only provides suggestions for dimensional evaluation and partially for evaluating profile parameters of surface roughness. What needs to be added to the standard is the development of models and measurement procedures to assess, among other things, the geometrical deviations and the surface roughness parameters. The procedures presented in the standard also need to consider the problems that can arise when digitizing a model manufactured from photopolymer materials. It is also crucial to pay attention to the development of procedures in terms of optical measurement methods, which are increasingly used in assessing the geometric accuracy and surface roughness of models made by additive methods [21,22,42]. So far, the development of specific guidelines and standards in the literature needs to be improved, as is the case with contact-based coordinate measuring systems.

During this research study, a test model was developed based on a literature review to assess the accuracy of PolyJet model manufacturing. The model, designed by the authors, used three types of photopolymer resins to develop and test measurement procedures. Measurements were then taken, and strategies were developed based on the produced model. Results were obtained to evaluate the geometry accuracy and surface roughness of models manufactured using the PolyJet method, utilizing optical coordinate measuring systems.

## 2. Materials and Methods

**Figure 1:**
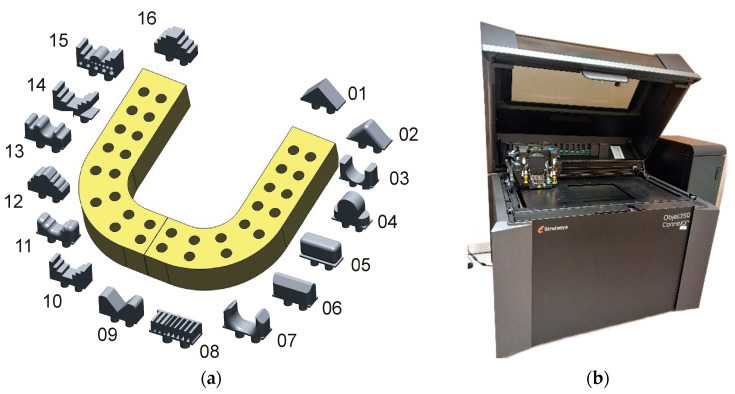
The process of designing and manufacturing models: (**a**) model with modular structure; (**b**) Object 350 Connex3 3D printer.

The test models were created using Geomagic X Design 12 software. Sixteen models and a component for positioning them during measurement were developed (Figure 1a). The model has a modular structure, and the positioning model has thirty-two slots on its surface for securing the 16 elements. The triangle side’s chord deviation and maximum edge length were set to 0.005 mm to minimize errors in the geometry data export to the stereolithography (STL) file. Additionally, the binary storage format was chosen over the American Standard Code for Information Interchange (ASCII) to reduce file size. The selected parameters in the tessellation process ensured that the accuracy of the 3D STL models was at least an order of magnitude higher than that of current 3D printers. This was done to prevent errors at the tessellation stage from being reproduced in the model production stage. The models were manufactured using the PolyJet method, which produces models from liquid, photo-curable polymer materials [8,9]. This research used Stratasys’s Object 350 Connex3 3D printer (Stratasys, Eden Prairie, MN, USA) (Figure 1b).

**Figure 2:**
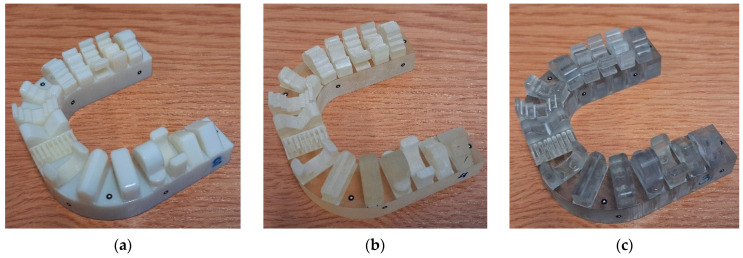
View of manufactured models made of the following materials: (**a**) Digital ABS Plus (**b**) RGD 720; (**c**) Vero Clear.

The entire manufacturing process for the models, utilizing the PolyJet method, took place within an enclosed space. First, the liquid resin material was heated to 30–60 °C to achieve its ideal viscosity. The high-quality mode was chosen to balance manufacturing speed and model precision. This mode allowed for the creation of the model with a layer thickness of 0.016 mm. Additionally, the matte mode was selected to improve the quality of the model surface. During manufacturing, thin layers of acrylic resin were continuously applied and then cured by exposure to an ultraviolet lamp. This approach eliminated the need for additional model exposure, which other technologies require. Materials were dispensed from print heads moving in the x-axis and y-axis directions. Some print heads dispensed the model material, while others dispensed the support material. The model material reproduced the model’s cross-section, and the remaining space was filled with support material. After each layer was completed, the platform was lowered by a specific layer thickness along the z-axis, and the next portion of the material was spread. Each layer was cured with UV light and combined with the previous layer to form the entire model. The Object Studio 7.1 software was used to prepare the digital files for 3D printing. The models were manufactured using three types of photopolymer resins: Digital ABS Plus (referred further as ABS) (Figure 2a), RGD 720 (referred further as RGD) (Figure 2b), and Vero Clear (referred further as VC) (Figure 2c). After manufacturing, the support material was removed mechanically using a pressure washer.

**Figure 3:**
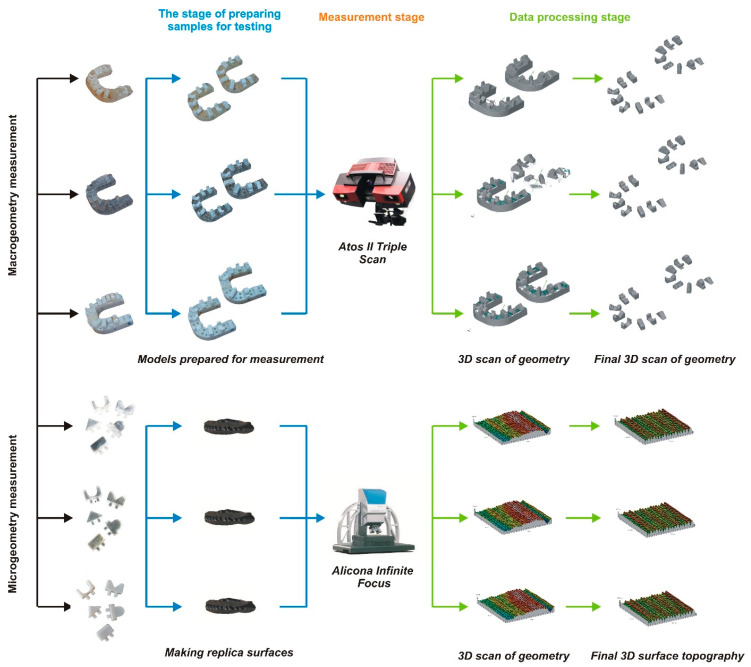
The overall scheme of the adopted methodology for measuring macro- and micro-geometry.

In the next step, the process of preparing the models to measure macro- and micro-geometry began. The entire procedure is depicted in Figure 3.

The Atos II Triple Scan system was used in the micro-geometry measurement process. This system is based on illuminating the object with structured light. The system consists of a tripod on which the measuring head is placed. It has a projector and two cameras with a resolution of 5,000,000 pixels. In addition, the system includes a rotary table with a computer system that processes the measurement data. Before measurement, the model was coated with matting powder to account for surface reflectivity. While using a scanner, information about the spatial position of points was captured based on the principle of triangulation. This involved calculating the intersection point in the space of the plane formed by the structured light beam and the ray from the center of the camera pixel. The raster image, deformed on the object’s surface, underwent computer analysis to produce direct data representing a three-dimensional point cloud. A rotary table was used to fully visualize the 3D data of the model’s geometry, with measurements taken every 22.5 degrees. After each table rotation, the measured data were combined to create the final 3D visualization of the model. To better represent the geometry of the 16 test models qualitatively, two measurements were made while maintaining repeatable data acquisition conditions. The first eight elements were measured in the first and the rest in the second. In the next step, the models obtained during the measurement were saved in STL format and subjected to further processing. This consisted of removing the model’s geometry to position the elements during measurement. As a result, 16 different model geometries were developed. However, the models numbered 08 and 15 could not be measured sufficiently and were therefore not taken for macro-geometry assessment.

**Figure 4:**
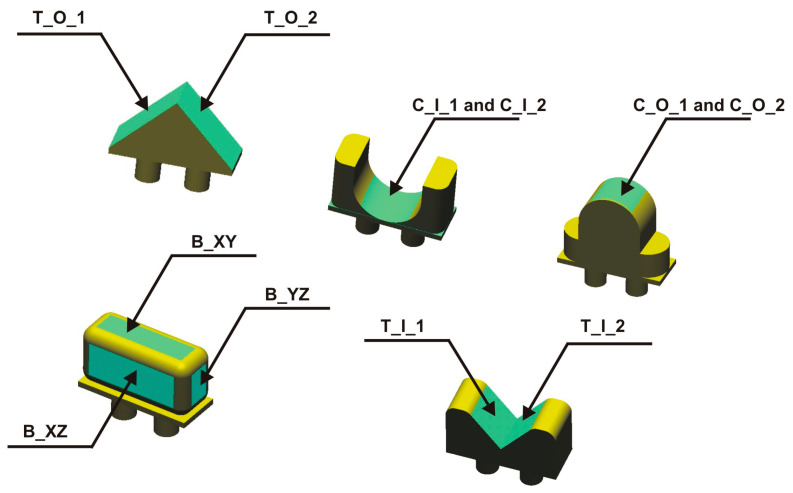
Selected models with indications of the measurement areas for evaluating micro-geometry.

The surface topography was measured using Alicona’s InfiniteFocusG4 focus variation microscope. A low depth of field characterizes the optical system of the microscope. Based on the z-axis scanning, a maximum on the focus curve for each pixel and its surroundings is determined. Based on this, a given pixel can be assigned a z-coordinate value. An objective with a magnification of ×20 was used during the conducted tests. The measured area was 0.54 mm × 4.34 mm. The measurement area’s longer side was perpendicular to the direction of the prevailing irregularities. The pixel size was 0.44 µm × 0.44 µm, and the vertical resolution was 0.1 µm. Processing of the measurement data and determination of the topography parameters was carried out in SPIP 6.4.2. Data processing included reducing peak artifacts, cropping the area to 0.54 mm × 4.00 mm, and removing the shape with a third-degree polynomial function. Surface topography parameters were determined from the primary profile. In evaluating the micro-geometry, models numbered 01 and 09 (triangular geometry—T), 03 and 04 (cylindrical geometry—C), and 05 (rectangular block geometry—B) were selected. The models and measuring surfaces are marked in Figure 4.

## 3. Results

### 3.1. Macro-Geometry Analysis

**Figure 5:**
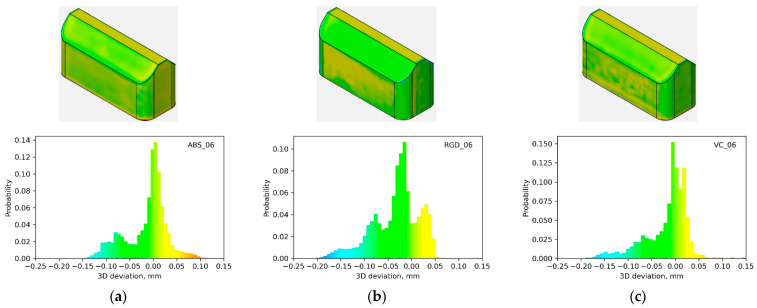
Local deviation maps and histograms for model 06 made of (**a**) Digital ABS Plus; (**b**) RGD 720; (**c**) Vero Clear material.

**Figure 6:**
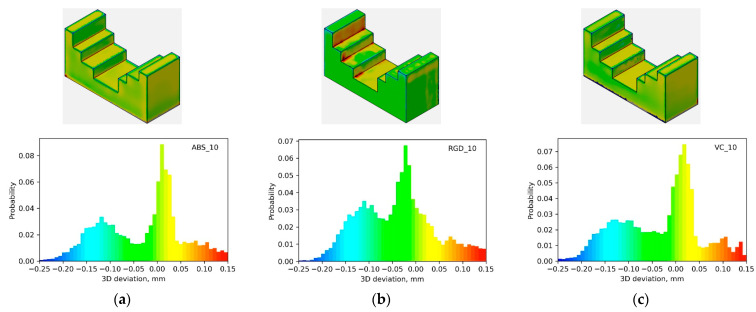
Local deviation maps and histograms for model 10 made of (**a**) Digital ABS Plus; (**b**) RGD 720; (**c**) Vero Clear material.

**Figure 7:**
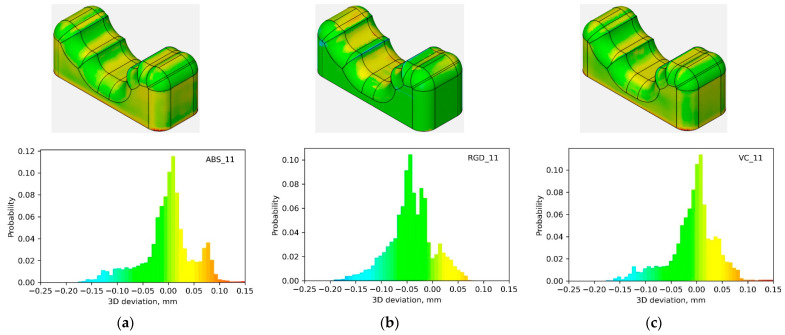
Local deviation maps and histograms for model 11 made of (**a**) Digital ABS Plus; (**b**) RGD 720; (**c**) Vero Clear material.

Macro-geometry analyses were conducted on 14 models from three photopolymer resins, including Digital ABS Plus, RGD 720, and Vero Clear. The analyses relating to the macro-geometry assessment were carried out using GOM Inspect 2019 software. Local deviations from the nominal geometry were assessed for each model. Initially, color deviation maps and histograms of deviations were examined. It was observed that regardless of the material used, the nature of the histogram trends for a specific model geometry remained consistent. The results for three example geometries are illustrated in Figure 5, Figure 6 and Figure 7. From the shape of the histograms, it can be seen that the progression of the deviations is related to the given geometry, i.e., regardless of the material, problems with the representation of the nominal geometry appear at the exact locations. Negative deviations were observed on convex edges, while positive ones were on concave edges. Figure 5 (for model 10) demonstrates this most clearly. Moreover, relatively large positive deviations were also found at the bottom edges of the models.

**Figure 8:**
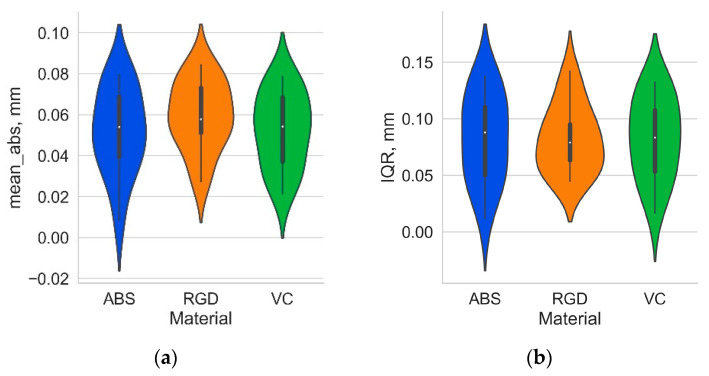
Violin plot presenting distribution of (**a**) mean absolute value; (**b**) interquartile range of local deviations for the 14 types of geometry analyzed depending on the sample material.

**Figure 9:**
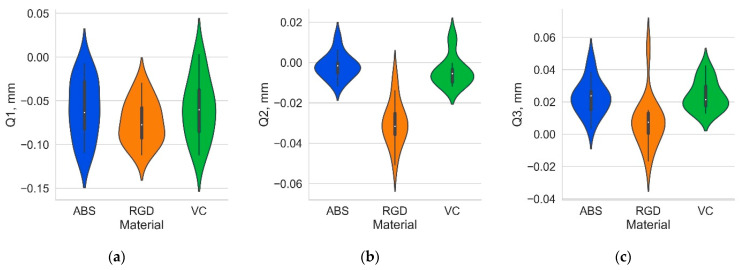
Violin plot presenting distribution of (**a**) first quartile, (**b**) median, and (**c**) third quartile for the 14 types of geometry analyzed depending on the sample material.

However, the distributions for the Vero Clear and Digital ABS Plus material models are more similar for a given geometry. Descriptive statistics analyses confirmed the high geometry similarity (suggested by analysis of histograms) between Digital ABS Plus and Vero Clear material models. As previously mentioned, a set of 3D local deviations was determined for each sample. The mean of the absolute deviations (mean_abs), first quartile (Q1), median (Q2), third quartile (Q3), and the interquartile range (IQR) of deviation values were then determined for each sample. The distributions of these statistics by material type are shown in Figure 8 and Figure 9, and the mean values and standard deviation for the 14 samples analyzed are summarized in Table 1.

To test whether the sample material had a statistically significant effect on the descriptive statistics obtained, a one-way analysis of variance (one-way ANOVA) was performed for paired data (the part number was the subject identifier). The normality of the distribution within groups was checked beforehand with the Shapiro–Wilk test. Equality of variance within groups was checked using the Bartlett test (if the data came from a population with a normal distribution) or the Levene test (if the data in any group did not come from a population with a normal distribution). If the result of the variance analysis indicated a significant effect on the material, post-hoc tests were performed (also for paired data). A significance level of 0.05 was assumed for all statistical tests. The results of the ANOVA are presented in Table 2, and the post-hoc tests in Table 3.

Table 2 shows that, of the statistics analyzed, only the value of the interquartile range was not dependent on the type of sample material and was approximately 0.08 mm (Table 1). The failure to reject the null hypothesis adopted in the analysis of variance for IQR indicates that the more significant variation in deviations expressed by IQR was due to the change in geometry analyzed rather than the material used.

The mean absolute deviation, median, and quartile values of the three samples from Digital ABS Plus and Vero Clear were statistically equal, while the parameters determined for the RGD 720 material differed. According to Table 1, the RGD 720 material had a higher mean absolute deviation (0.059 mm), about 13% higher than the other two materials (0.052 mm). Overall, considering all three materials, it can be stated that the average for the 14 models mean absolute deviations were in the range of 0.05–0.06 mm. Analyzing the effect’s strength using the common language effect size (CLES), the most significant differences between the materials are reflected in the median value Q2, followed by Q3 and mean absolute deviation (a more substantial effect strength is observed with CLES values higher than 0.5). The average median value for all materials was negative, which indicates the dominance of negative deviations. For Digital ABS Plus and Vero Clear materials, however, the median is statistically equal to 0; therefore, it can be assumed that the number of negative and positive deviations is similar for those materials. For RGD 720, the median value is noticeably smaller at −0.030 mm. For this material, on the other hand, Q3 is statistically equal to 0. The values of Q2 and Q3 for the RGD 720 material are about 0.02–0.03 smaller than for the other two materials. For Digital ABS Plus and Vero Clear materials, 50% of the central deviations (between Q1 and Q3) lie within the range (−0.06, 0.03 mm) and for RGD 720 material within the range (−0.08, 0.01 mm). Figuratively speaking, it can be said that in the case of RGD 720, the produced models were “smaller” than those of Digital ABS Plus and Vero Clear, which may indicate a more significant shrinkage of this material. In Q1, a statistical difference was shown only between the RGD 720 and Vero Clear samples (for the Vero Clear material, the mean Q1 value was more significant than RGD 720). However, it should be noted that for the RGD 720 and Digital ABS Plus pair, the *p*-value (Table 3) is only slightly bigger than the accepted significance level.

### 3.2. Micro-Geometry Analysis

**Figure 10:**
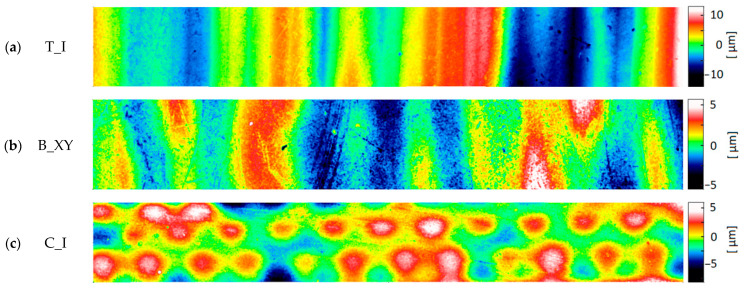
Typical topography maps of samples made of Digital ABS Plus material: (**a**) triangular sample (T_I); (**b**) block sample (B_XY); (**c**) cylindrical sample (C_I) (area 0.54 mm × 4.00 mm).

**Figure 11:**
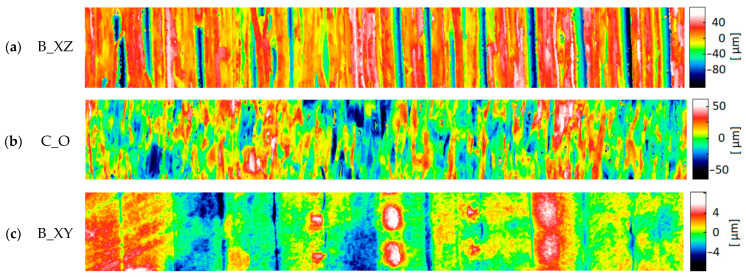
Typical topography maps of samples made of RGD 720 material: (**a**) block sample (B_XZ); (**b**) cylindrical sample (C_O); (**c**) block sample (B_XY) (area 0.54 mm × 4.00 mm).

**Figure 12:**
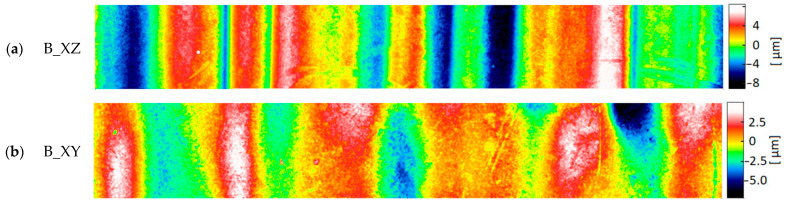

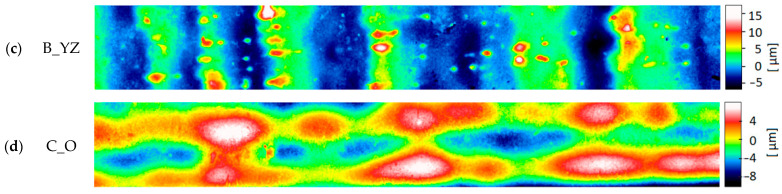
Typical topography maps of samples made of Vero Clear material: (**a**) block sample (B_XZ); (**b**) block sample (B_XY); (**c**) block sample (B_YZ); (**d**) cylindrical sample (C_O) (area 0.54 mm × 4.00 mm).

The most commonly analyzed surface topography parameters are Sa (arithmetic mean height) and Sz (maximum height). The topography was measured using an optical method prone to peak artifacts. For this reason, the sum of the Abbot–Firestone curve parameters Spk + Sk + Svk (Spk—reduced peak height, Sk—core height, Svk—reduced dale depth) was taken as an indicator of the total height of roughness. Most of the measured surfaces were characterized by a dominant structure direction. Figure 10, Figure 11 and Figure 12 show the surfaces representing the different types of structure measured on the samples of a given material. Within a given material, they are ordered from clearly directional (Figure 10a, Figure 11a and Figure 12a) to more isotropic surfaces (Figure 10c, Figure 11c and Figure 12c,d). The topographies of the samples made of Digital ABS Plus and Vero Clear material were similar. They mainly consisted of bands of peaks and pits of considerable width. For only 1–2 surface types, a different pattern of hills was observed, where they have a more oval shape (Figure 10c and Figure 12d). Much smaller peaks similar to ‘lumps’ are also sometimes observed on Vero Clear samples (Figure 12c).

**Figure 13:**
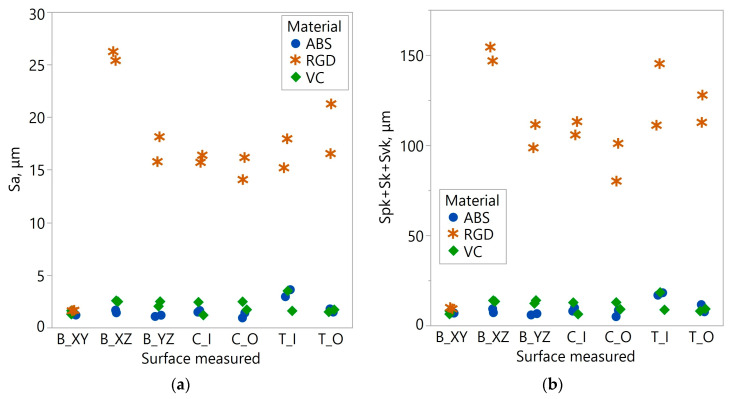
Values of the parameter: (**a**) Sa; (**b**) the sum of Spk + Sk + Svk measured on different samples.

The topography of the RGD 720 samples was significantly different. In all topographies, apart from the image of sample B_XY, bands of peaks and pits with relatively small widths (Figure 11a,b), compared to Digital ABS Plus and RGD 720 samples, were visible. These bands were also characterized by greater steepness of slopes and higher peaks/deeper pits. The differences between the topographies of the RGD 720 samples and those of the other materials are expressed in the Sa and Spk + Sk + Svk parameters (Figure 13). The average Sa values for the Digital ABS Plus and Vero Clear samples were approximately 1.6 and 2.0 µm, respectively. In comparison, for RGD 720, it was 15.9 µm. The total roughness height expressed by Spk + Sk + Svk for the Digital ABS Plus and Vero Clear samples was approximately 9.1 and 10.5 µm, respectively, while for the RGD 720, it was 101.9 µm. The average values registered for the RGD 720 samples are 8–10 times higher than for Digital ABS Plus and Vero Clear (Table 4).

The exception is sample B_XY, where similar values were observed regardless of material. The B_XY surface represents only one final manufactured layer of the model. For Digital ABS Plus and Vero Clear samples, analyses of variance were carried out for paired data (the type of geometry was a random effect), testing whether the type of material influences the values of the parameter Sa and the sum of Spk + Sk + Svk. The probability determined in these tests was more significant than the accepted significance level (the *p*-value for the dependent variable Sa was equal to 0.067 and 0.131 for Spk + Sk + Svk. Statistically, the mean values of the tested surface roughness parameters of the Digital ABS Plus and Vero Clear samples were equal. The variability of the analyzed values resulting from the different measurement locations expressed by the coefficient of variation CV was relatively high. For the RGD 720 and Digital ABS Plus samples, it was about 43–45%, and for the Vero Clear, it was about 32%. In the case of RGD 720, a significant contribution to the CV value came from the two values recorded for the B_XY sample. Without these two values, the coefficients of variation for the RGD 720 samples would have more than halved.

## 4. Discussion

With the PolyJet method, the machine manufacturer sets almost all process parameters automatically. Operators can only change, among other things, the layer thickness [40], the surface finishing setting (e.g., matte or glossy) [43], and the model’s orientation in the 3D printer space [44,45]. These parameters significantly influence the macro- and micro-geometry of the models produced. Currently, in the literature, there are many items concerning the metrological evaluation of models made using the PolyJet method. They are mainly concerned with dimensional evaluation and partly with form deviations, e.g., flatness and cylindricity. The dimensional assessment was most often carried out using a digital caliper [46], a coordinate measuring machine [47,48,49], and an optical microscope [49]. Research on geometry accuracy is also emerging, particularly concerning gear models [50] and anatomical structures [51,52,53] using optical measurement systems. What is missing in this research, however, is the presentation of the studies in a broader scope, i.e., involving a larger group of measurement elements, guidance on the procedures of the measurement procedures themselves, and the presentation of a wider spectrum of analyses in the field of macro-geometry studies. Considering the results of the geometric accuracy assessment in these publications, the results were determined based on RGD 720, Vero Dent, Full Cure 830, and Vero Clear material models. In the case of the RGD 720 material from which the gear wheel was manufactured, the results are within a deviation range of ±0.070 mm. In the case of the tooth crown models made from the Vero Dent material, the deviation results are within a range of 0.07 mm ± 0.014 mm. In the case of the mandibular model made from the Full Cure 830 material, the deviations are within a range of ±0.15 mm, and in the case of the complete-arch model made from Vero Clear material, the deviations are within a range from −0.06 mm to 0.15 mm. The results presented in the publication [50] for manufacturing the gear model from the RGD 720 material are similar to those presented in this article. However, when comparing the results obtained with the Vero Clear material [53], they differ from those in this article. The reason for the difference in the results obtained may be due to the fact that in the publication [53], only one geometry type was analyzed. In the case of our study, we present a broader investigation, the results of which were obtained by averaging data collected from as many as 16 different types of geometry. It is also difficult to compare the results obtained with the Digital ABS Plus material, as no such results have been developed.

Surface roughness measurements are most often carried out using the contact method, and the results are processed based on data obtained from a single measured profile [43,44,45,54]. The above-mentioned publications mainly investigated the effect of the surface finishing setting on surface roughness quality [43,44,45]. Some also investigated the roughness on different surfaces of manufactured models [54]. RGD 720 [43], Full Cure 830 [48], and Full Cure 720 [44,45,54] materials were tested. Only publication [48] used an optical profilometer to verify surface roughness parameters. In the publication [48], a study was carried out on changing the orientation of the model in the 3D printer space. In the process of manufacturing the model, Vero White material was used. It was noted in publication [48] that the smallest values of the Sa and Sq parameters were obtained when the model was manufactured vertically. The average value of the Sa parameter for the manufactured surface along the print layers was 0.95 µm, and on the last print layer 1.14 µm. Considering the averaged Sa value obtained for the Vero Clear material (which was approximately 2 µm), it is higher than the Sa value for the Vero White material reported in [48]. This difference may be due to the fact that the value developed in the current publication was based on averaging the results of a larger number of test models, which also differed in the type of geometry. At this point, the development of surface roughness results using the optical method for the Digital ABS Plus material has received no attention in the publications. In the case of roughness for the RGD 720 material, only the value of the Ra parameter was assessed in the publication [44]. It ranged from 0.03 µm to 13.59 µm.

## 5. Conclusions

As functional models of polymeric materials are increasingly being made using additive techniques, it is essential to develop metrological results to assess surface texture quality. This aspect is crucial, as the appropriate forming of the macro- and micro-geometry directly affects the model’s tightness, accuracy, fit, wear, or deformation. With the developed test models and measurement procedures, statistical results were obtained to evaluate the geometric accuracy and surface roughness of models made of three types of photopolymer resins using the PolyJet method. Based on these, it can be concluded that, in macro-geometry, the average for the 14 models mean absolute deviations were in the range of 0.05–0.06 mm. Negative deviations predominated in the RGD 720 models. The number of negative and positive deviations is comparable for the Digital ABS Plus and Vero Clear models. Approximately, for Digital ABS Plus and Vero Clear materials, 50% of the central deviations (between Q1 and Q3) is in the range of (−0.06, 0.03 mm), and for RGD 720 material, in the range of (−0.08, 0.01 mm). The more significant variation in the deviations in the models expressed by the IQR parameter was more due to the change in the analyzed geometry than to the material used. In terms of micro-geometry, the structure is directional on most of the measured surfaces. Statistically, the mean values of the parameter Sa and the sum of Spk + Sk + Svk of the Digital ABS Plus and Vero Clear samples were equal. For these materials, the average Sa values were 1.6 and 2.0 µm, and the Spk + Sk + Svk values were 9.1 and 10.5 µm, respectively. The observed topography maps of the samples made from these materials are similar. Mostly, the maps of the RGD 720 samples are markedly different from those of the Digital ABS Plus and Vero Clear samples. In RGD 720 samples, one can observe irregularities with much steeper slopes. The widths of the bands of peaks and pits are smaller than those of the samples from the other two materials. The average values of the Sa parameter and the sum of Spk + Sk + Svk were 8–10 times higher than for the Digital ABS Plus and Vero Clear samples.

The research findings presented here are a foundation for future studies, such as developing procedures to improve models’ geometry accuracy and surface roughness. Further research is needed to explore the mechanical properties of models produced using the PolyJet method. This could lead to the creation of model sets for casting molds and the direct production of casting molds for manufacturing small sets of casting parts used in the automotive, aerospace, and medical industries.

## 6. Patents

The result of the procedure presented in this manuscript is the granting of a patent: “A model for medical applications and how to manufacture a model for medical application” by the Patent Office of the Republic of Poland. Application number P.434490, exclusive correct number: Pat.242932.

## Figures and Tables

**Table 1:** Mean value (mean) and standard deviation (std) of statistical parameters obtained for the 14 types of geometry analyzed depending on the sample material.

Material	Mean Absolute Deviation Mean_abs, mm		First Quartile Q1, mm		Median Q2, mm		Third Quartile Q3, mm		Interquartile Range IQR, mm	
	Mean	Std	Mean	Std	Mean	Std	Mean	Std	Mean	Std
Digital ABS Plus	0.052	0.021	−0.060	0.034	−0.002	0.006	0.023	0.011	0.083	0.039
RGD 720	0.059	0.017	−0.074	0.025	−0.030	0.011	0.008	0.016	0.082	0.030
Vero Clear	0.052	0.018	−0.059	0.035	−0.004	0.008	0.024	0.009	0.083	0.036

**Table 2:** Results of the ANOVA testing the material’s influence on the obtained descriptive statistics of local deviations.

Dependent Variable	*p*-Value
Mean absolute deviation (mean_abs)	0.002
Interquartile range (IQR)	0.998
First quartile (Q1)	0.016
Median (Q2)	2.07 × 10^−10^
Third quartile (Q3)	4.21 × 10^−04^

**Table 3:** Results of post-hoc tests investigating the influence of the material on the obtained descriptive statistics of local deviations.

Dependent Variable	Contrast	A	B	*p*-Value	Common Language Effect Size (CLES)
Mean absolute deviation (mean_abs)	Material	Digital ABS Plus	RGD 720	0.011	0.40
		Digital ABS Plus	Vero Clear	0.758	0.52
		RGD 720	Vero Clear	0.006	0.60
First quartile (Q1)	Material	Digital ABS Plus	RGD 720	0.054	0.63
		Digital ABS Plus	Vero Clear	0.831	0.49
		RGD 720	Vero Clear	0.013	0.38
Median (Q2)	Material	Digital ABS Plus	RGD 720	3.44 × 10^−06^	0.99
		Digital ABS Plus	Vero Clear	0.359	0.65
		RGD 720	Vero Clear	3.41 × 10^−08^	0.03
Third quartile (Q3)	Material	Digital ABS Plus	RGD 720	0.006	0.87
		Digital ABS Plus	Vero Clear	0.765	0.51
		RGD 720	Vero Clear	0.001	0.10

**Table 4:** Descriptive statistics of surface topography parameter values depending on the material sample.

Parameter	Statistics	Material		
		Digital ABS Plus	RGD 720	Vero Clear
Arithmetical mean height (Sa)	mean, μm	1.63	15.86	1.96
	median, μm	1.43	16.27	1.69
	std, μm	0.74	7.06	0.62
	CV, %	45.3	44.5	31.65
Reduced peak height (Spk) + core height (Sk) + reduced dale depth (Svk)	mean, μm	9.10	101.88	10.51
	median, μm	7.74	111.21	9.04
	std, μm	3.92	44.10	3.40
	CV, %	43.1	43.29	32.33

## Data Availability

The original contributions presented in the study are included in the article, further inquiries can be directed to the corresponding author.
